# Strategy for a globally coordinated response to a priority neglected tropical disease: Snakebite envenoming

**DOI:** 10.1371/journal.pntd.0007059

**Published:** 2019-02-21

**Authors:** David J. Williams, Mohd Abul Faiz, Bernadette Abela-Ridder, Stuart Ainsworth, Tommaso C. Bulfone, Andrea D. Nickerson, Abdulrazaq G. Habib, Thomas Junghanss, Hui Wen Fan, Michael Turner, Robert A. Harrison, David A. Warrell

**Affiliations:** 1 Australian Venom Research Unit, Department of Pharmacology and Therapeutics, University of Melbourne, Parkville, Victoria, Australia; 2 Department for the Control of Neglected Tropical Diseases, World Health Organization, Geneva, Switzerland; 3 Chittagong Medical College Hospital, Chittagong, Bangladesh; 4 Alistair Reid Venom Research Unit, Parasitology Department, Liverpool School of Tropical Medicine, Pembroke Place, Liverpool, United Kingdom; 5 California Academy of Sciences, San Francisco, California, United States of America; 6 Department of Medicine, College of Health Sciences, Bayero University Kano, Kano, Nigeria; 7 Section Clinical Tropical Medicine, University Hospital Heidelberg, Heidelberg, Germany; 8 Divisão Bioindustrial, Instituto Butantan, São Paulo, Brazil; 9 Infection and Immuno-Biology, Wellcome, London, United Kingdom; 10 Nuffield Department of Clinical Medicine, University of Oxford, United Kingdom; Universidad de Costa Rica, COSTA RICA

In one of his final essays, statesman and former United Nations secretary general Kofi Annan said, ‘Snakebite is the most important tropical disease you’ve never heard of’ [1]. Mr. Annan firmly believed that victims of snakebite envenoming should be recognised and afforded greater efforts at improved prevention, treatment, and rehabilitation. During the last years of his life, he advocated strongly for the World Health Organisation (WHO) and the global community to give greater priority to this disease of poverty and its victims.

Snakebite envenoming (SBE) affects as many as 2.7 million people every year, most of whom live in some of the world’s most remote, poorly developed, and politically marginalised tropical communities [2]. With annual mortality of 81,000 to 138,000 and 400,000 surviving victims suffering permanent physical and psychological disabilities, SBE is a disease in urgent need of attention [2–4]. Like many diseases of poverty, SBE has failed to attract requisite public health policy inclusion and investment for driving sustainable efforts to reduce the medical and societal burden. This is largely due to the demographics of the affected populations and their lack of political voice [5].

## Devising a consensual pathway to the goal of halving deaths and disability by 2030

Despite decades of concern over the impact of SBE in low-middle-income countries (LMICs), a lack of any clear mandate from member states has made it difficult for WHO to take substantial action [4, 6–9]. Indeed, it wasn’t until 2015 when alarm over the possible therapeutic vacuum in Africa, caused by Sanofi-Pasteur’s decision to cease production of their FAV-Afrique antivenom, galvanised renewed calls for urgent action [9, 10]. In 2017, after intense advocacy by concerned stakeholders including Médecins Sans Frontières [10, 11], the Global Snakebite Initiative [5, 12–14], Health Action International, and a detailed submission by more than 20 countries, WHO listed SBE as a priority neglected tropical disease (NTD) [15, 16]. In May 2018, the 71st World Health Assembly adopted a robust resolution (WHA71.5) on SBE, providing WHO with a strong mandate to take action [17].

The inclusion of SBE in the WHO NTD portfolio donates powerful attention to this disease. Even before the resolution was adopted, WHO’s Department of the Control of Neglected Tropical Diseases had already established a 28-member SBE Working Group (SBE-WG) to support WHO in drafting a road map to implement strategies to prevent, reduce, and control the snakebite burden. In June 2018, WHO convened a Wellcome-hosted meeting of the SBE-WG to review a first draft of the road map document.

**Fig 1:**
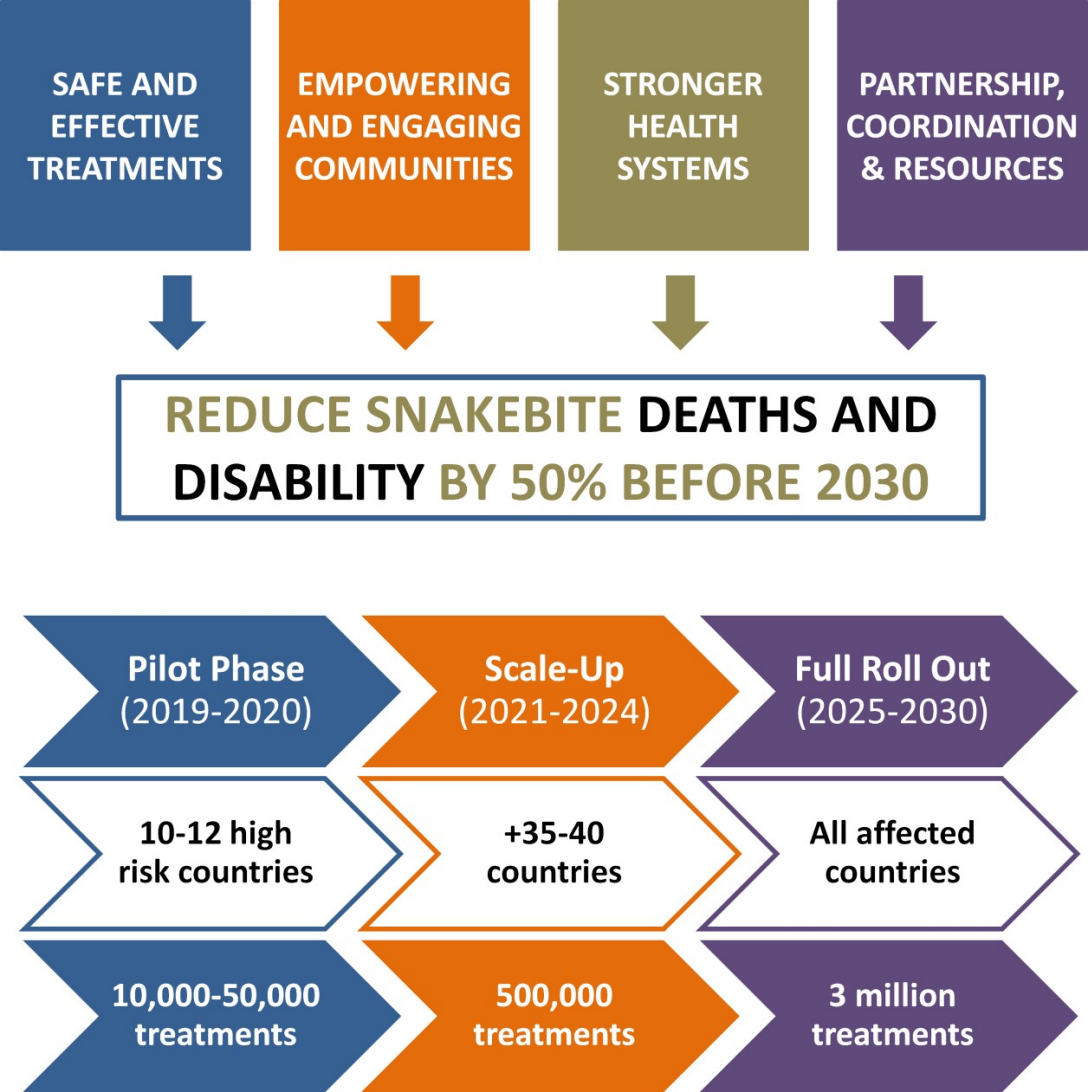
Summary of WHO snakebite envenoming road map objectives, impact goals, and timeline phases.

Central to the design of this strategic plan is the ambitious goal of halving the deaths and disability caused SBE by 2030 (Fig 1). The consensus of the SBE-WG was that implementing an integrated program based on building capacity and directing response to snakebite-affected regions offers the most effective approach to achieving this goal. Rather than risk this initiative being perceived as a standalone issue, the SBE-WG considered that efforts to combat SBE need to be incorporated within national and regional health plans and aligned with global commitments to achieving universal health coverage and the Sustainable Development Goals (SDG). With this in mind, four key pillars (Fig 1; Table 1) have been prioritised:

1. Ensuring that safe and effective treatment is accessible and affordable for all;
2. Empowering regional, national, and local communities to take proactive action;
3. Strengthening health systems to deliver better outcomes; and
4. Building a strong global coalition of partners to build advocacy, mobilise resources, coordinate action, and ensure that implementation of the roadmap is successful.

**Table 1:** Summary of overall program areas.

Objective	Safe and effective treatment	Empowering and Engaging communities	Stronger health systems	Partnership, coordination and resources
Key activities	Programme-wide resource mobilisation to support all WHO activities and work plans			
	Make safe, effective antivenoms available, accessible, and affordable to all	Active community engagement and participation	Strengthening community health services	Supporting governance and leadership
	Better control and regulation of antivenoms	Improve SBE prevention, risk-reduction and avoidance	Facilitating research and policy development around healthcare cost mitigation	Promoting advocacy, effective communication and productive engagement
	Prequalification of antivenoms	Effective prehospital care and ambulance transport	Improving infrastructure, services and health facilities	Enhancing integration, coordination and cooperation
	Integrated health worker training and education	Accelerate development of prehospital treatments	Country-level implementation via national and sub-national health plans	Build strong regional partnerships and alliances
	Improving clinical decision-making, treatment, recovery and rehabilitation	Improve health care-seeking behaviours	Enhanced disease burden monitoring and surveillance	Coordinated data management and analysis
	Investing in innovative research on new therapeutics	Build a strong understanding of socio-cultural and economic factors affecting outcomes	Research on SBE ecology, epidemiology, clinical outcomes and therapeutics	Research to build a strong and sustainable investment case

The scale of this challenge is considerable and its achievement requires a globally coordinated and implemented strategy, with the WHO best positioned to coordinate this effort.

### Safe and effective treatment

SBE is a medical emergency that particularly afflicts the world’s poorest people living in communities with the lowest quality of life indices [18]. The sooner a victim receives effective treatment, the greater the likelihood of a full recovery and an early return to normal life. For more than 120 years, the cornerstone of snakebite treatment has been the administration of animal-derived immunoglobulins (antivenoms) [2]. Most antivenoms are produced from the hyperimmune plasma of horses or sheep, and the methods used have changed very little in the last 50 to 60 years [19]. Consequently, the quality and safety of some antivenoms remain poor. Production inefficiencies, inadequate market demand, low manufacturing volumes, storage limitations, and distribution problems have combined with inadequate funding for procurement, poor health-worker training, and local bias towards traditional healing to create a fragile market at risk of collapse in many parts of the world [20]. These market fragilities explain why, for much of the last century, antivenom production has largely been the domain of public health laboratories, few of which have been adequately capitalised to keep pace with modern pharmaceutical manufacturing technologies. And while private manufacturers have gained an increasingly important role during the last four decades, especially in Asia and in Africa, the nature of the market limits their capacity to resource infrastructure and innovation [20, 21]. As a consequence, there has previously been little incentive for innovation or investment in new technology.

Comprehensively addressing these issues is a key priority for WHO. The SBE-WG, concerned by the current critical situation in sub-Saharan Africa, have set a target of delivering at least 500,000 effective antivenom treatments to that region each year by 2024. By the end of 2030, the target is to deliver, globally, 3 million effective regionally specific treatments per year. To achieve this, WHO will work to strengthen the production of antivenoms, improve regulatory control, and most importantly, rebuild and reinvigorate the market by ensuring that safe and effective products are available, accessible, and affordable.

WHO has already begun this process by undertaking a comprehensive product risk assessment for sub-Saharan Africa. Its results are expected to be published in early 2019. This evaluation included robust preclinical evaluation of products and site inspections to evaluate compliance with good manufacturing practice (GMP). The result will be a WHO-recommended list of products suitable for procurement across sub-Saharan Africa, providing purchasers and end-users with confidence that products are fit-for-use, and providing manufacturers with incentive to improve the quality of products and comply with GMP and other regulatory requirements as a pathway to restoring investment confidence in the market and generating income commensurate with sustained delivery. As funding becomes available, WHO plans to undertake similar antivenom risk–benefit assessments for other regions and to consider the introduction of antivenom prequalification as a tool for further strengthening the production of these life-saving drugs. Such a pathway will only be possible if technical barriers—such as the need to establish appropriate reference standards, minimum product design specifications, and pathways for acquiring robust clinical evidence—are resourced and overcome.

A range of other initiatives (Box 1) will also be implemented to reinvigorate investment in antivenom production and to establish an environment that attracts new manufacturers, stimulates research, and encourages innovation. WHO will take key lessons from highly effective vaccine stockpiling ventures, such as the Oral Cholera Vaccine Stockpile [22, 23], to model and then establish an African Antivenom Stockpile as a pathway to creating a stable supply of quality-assured WHO-recommended antivenom products in sub-Saharan Africa. This stockpile is designed to reshape the current market: converting it from one in which low production at high unit cost has driven weak demand and distribution that culminated in poor accessibility and affordability; to one in which there is equitable access and affordability because of higher production at lower cost as a result of increased market confidence, higher demand, improved procurement, and wider distribution.

**Box 1. Priority actions to ensure sustainable supply and accessibility of safe, effective, and affordable antivenoms**

**Resource actions**

- Identify and mobilise resources to reshape the market for sustained antivenom delivery;
- Galvanise funding for research to improve existing antivenom technologies;
- Prioritise investment in clinical research, including clinical trials of the safety and effectiveness of treatments;
- Increase resources to reduce treatment-associated patient financial and social vulnerability.

**Actions to improve quality, safety, and effectiveness of antivenoms**

- Global risk–benefit assessments of antivenom products to ensure that at least three quality-assured and fit-for-use antivenoms are accessible in each region;
- Strengthen the capacities of antivenom manufacturers to increase production, improve research and development, and meet GMP and quality control requirements and compliance with regulatory standards;
- Development and introduction of target product profiles (TPP’s), venom and antivenom reference standards, and an appropriate prequalification pathway;
- Provide essential guidance, regulatory and technical support to National Regulatory Authorities (NRAs), National Control Laboratories (NCLs), and National Health Authorities (NHAs) to strengthen and build capacity for effective regulation of antivenoms in all regions;
- Stimulate enhanced collaboration between research and manufacturing sectors to improve all aspects of antivenom design, production, quality control, and evaluation.

**Actions to increase accessibility and affordability of antivenoms**

- Establish an antivenom stockpile programme initially for countries in sub-Saharan Africa;
- Working with countries, partners and donors, apply a range of initiatives (in addition to establishing revolving stockpiles) to reshape regional antivenom markets, increase confidence, incentivise demand, and expand the availability, accessibility, and affordability of WHO-recommended antivenoms;
- Deliver cost-mitigation and financing schemes to ensure access to effective treatment and healthcare.

**Actions to ensure long-term sustainability of antivenom supply**

- Galvanise LMIC countries to support investment in local antivenom manufacturing;
- Work with communities to improve health-care seeking behaviours, and with governments to support health worker training and education around the use of antivenoms.

The SBE-WG also agreed that there needs to be a range of actions to encourage increased collaboration between academia, clinicians, and industry to improve potency, specificity, and safety of current antivenoms, with an additional focus on development of new treatments. Research involving robust preclinical and clinical evaluation of antivenoms and other treatments for SBE will be encouraged, with clinical research prioritised for funding investment along with identifying sentinel sites where clinical trials can be conducted to high standards. Refining preclinical models to improve their reliability and relevance, and wider adoption of the 3R’s (reduction, refinement, and replacement) relating to the use of experimental animals in the production and testing of antivenoms are research priorities. With promising preclinical research on new therapeutic solutions to SBE management emerging, the need for investment in next generation therapies and diagnostics also needs funding support [24, 25]. The WHO will work with antivenom manufacturers, with national regulatory agencies, and with ministries of health to build capacity to ensure that all treatments for SBE are properly controlled and regulated.

The SBE-WG also concurred that any effort to control the SBE burden requires broader efforts at improving the overall management of snakebite victims. It is essential that standard approaches be developed and implemented across all tiers of health systems. There should be clear criteria for judging the success (or otherwise) of treatment. Currently there is little assistance or support available to SBE survivors suffering residual disability. Establishment of dedicated rehabilitation programs, addressing both psychological and physical disability, will improve recovery of survivors, enabling more of them to return to useful, productive lives, therefore increasing economic productivity.

## Empowering and engaging communities

As well as effective engagement with health decision makers at regional and national levels, there is strong evidence linking the success of disease interventions to engagement with local communities to engender trust in outcomes and hence productive participation [26–28]. A major barrier to improving the treatment of SBE is the perception across many LMIC communities that rather than being a physical illness amenable to medical treatment, snakebites, like many other unexpected illnesses, are associated with deity punishment, witchcraft, or other powerfully persuasive phenomena that are often very locally specific [29, 30]. Context-appropriate engagement with local communities is therefore important to overcome these misconceptions and create a balance between traditional customs and modern healthcare. It is equally vital that local hospitals and/or health centres are equipped with effective, affordable, and safe antivenom. Funding should be made available to study the human–snake conflict interface, the social and cultural barriers to allopathic medicine, and the development and deployment of effective prehospital care interventions that can improve ‘first mile’ care and sustain life. WHO will propose engagement with local champions who can lead efforts to introduce acceptable prehospital ‘first aid’, encourage earlier presentation to primary health care centres, facilitate safe transport, and provide basic life-support. Accelerating preclinical and clinical testing of promising prehospital adjunctive treatments, such as the phospholipase A_2_ inhibitor, Varespladib, as part of the WHO SBE research agenda may lead to early improvements in prehospital survival [25]. Coupled with improved training of primary healthcare workers in emergency treatment of SBE, safe referral of envenomed patients, and better access to basic life support commodities, antivenom, and adjunctive medicines, there is great potential to save lives in even the most remote settings. In many settings, community-level training about basic airway protection and safe transport to healthcare could save thousands of lives. The WHO road map recommends strong local engagement with communities to promote prevention, safe prehospital care, and improved healthcare-seeking behaviour combined with participation by traditional healthcare providers within the health system rather than outside it. This would mirror similar approaches that have been applied in some settings for other diseases such as Buruli ulcer and malaria [31, 32].

## Stronger health systems

Many of the components of a functional and responsive health system needed to improve the outcomes for snakebite victims are no different to those that improve access to universal healthcare for all people. Strengthening healthcare capacity and performance at community and higher national levels is vital and integral to achieving UHC2030 [33]. There is good evidence that such activities can have a substantial impact on the health of women and children, two groups who are vulnerable to poor outcomes after SBE due to reduced access and other factors [34]. Myanmar communities identified improved healthcare accessibility to antivenoms and greater affordability of health care as key priorities [35]. In Nepal, rapid access to healthcare positively improved outcomes after SBE [30, 36]. The SBE-WG agreed that integrated steps that build capacity of healthcare systems to better manage SBE and other diseases should be prioritised and that synergies should be identified and exploited to advance progress towards achieving SDGs for health and moving closer to UHC2030. The components that are directly relevant to snakebite victims range from access to prehospital care and ambulance transport through to effective diagnosis, the availability in hospital of essential medicines (including antivenoms), consumables and medical services (emergency, intensive care, radiology, pathology, renal care, paediatrics, surgical, etc.), complemented by rehabilitation and recovery support. There was strong agreement that increasing access to clear guidelines that standardise the diagnosis and treatment of snakebite patients and improving the training of doctors and other health workers was fundamental to ensuring better outcomes for patients. The road map calls for countries to increase training for all health workers in an integrated manner and to work towards improving infrastructure and resourcing of health facilities—steps that benefit entire communities.

One of the major difficulties associated with SBE is the relative paucity of high quality epidemiological surveillance data and the impact this has on being able to accurately report the burden of disease [2, 3]. Having access to accurate information, research data, and the results of surveillance is fundamental to health planning, monitoring, and assessment and is a key component of a strong health system and the elimination or control of NTDs [36, 37]. Globally, much needs to be done to improve the surveillance of SBE. Under the proposed road map, WHO will recommend inclusion of SBE as a ‘notifiable disease’. To support adoption of this designation, and to improve data quality and comparability, standardised clinical criteria adapted to specific regional needs will be developed and minimum data set definitions for community-acquired and hospital-acquired data introduced. A number of public health tools already used to control other diseases can be valuably adopted for building higher resolution systems to monitor progress to control SBE [38–40]. Research to develop and deploy new data-collection tools, or which broaden our knowledge of the health- and socio-economic impacts of SBE, the cost-benefit and cost-effectiveness of interventions, patient care financing, and effective monitoring and evaluation of road map progress is needed. The WHO will include SBE data in the Global Health Observatory (www.who.int/gho/) repository and will work with countries and partners to improve the collection, analysis, and reporting of surveillance data.

## Partnership, coordination and resources

Achieving the ambitious goal of reducing SBE mortality and disability by 50% by 2030 requires strong leadership from WHO, provision of requisite funding, identification and allocation of adequate resources, and the development of a dynamic global partnership to drive policy change, implementation, and evaluation of outcomes. Building a strong multidisciplinary and participatory collaboration is essential to increase effectiveness of interventions and mobilise resources to reduce the burden of SBE [41, 42]. Effective advocacy, built on robust data, will be vital to generate and mobilise the resources needed to implement the road map and to ultimately ensure the sustainability of the approaches being proposed. Stimulating research in priority areas where there are currently major gaps will ensure that appropriate tools are developed, and creating strategic partnerships will help ensure that research outcomes are effectively translated into new clinical and public health tools to reduce the burden of SBE.

The resolution on SBE (WHA71.5) passed at the 71st World Health Assembly in May 2018 robustly calls on countries to increase their efforts to prevent and control this disease, just as it requests that WHO also takes specific steps in this regard [17]. The WHO road map for SBE will set out pathways for the incorporation of this disease in regional- and country-level health plans and will focus on horizontal integration, complementary activities, and local stakeholder inclusion and participation. In-country and regional coordination mechanisms that integrate SBE with interventions for other diseases, such as wound care programmes for Buruli ulcer [43], promotion of footwear to prevent soil-associated diseases such as hookworm or podoconiosis [44], or the use of malaria bed nets (which can prevent nocturnal snakebites in places where people are sleeping) [45] will be promoted. Similarly, the success of programmes such as WASH that improve sanitation and human behaviours can help to reduce the risk of SBE [46].

## Next steps and key actions

The road map has been revised by the SBE-WG and will be shared with key stakeholders before it is published and officially launched in May 2019. WHO is preparing a budget for implementing resolution WHA71.5, which will require strong financial commitment from stakeholders. The roll-out of the road map is incremental, and as efforts scale up, the strategy will require increased investment to support expanded WHO activities and in-country implementation. Additional modelling of implementation costs and benefits of specific components of the strategy are being undertaken, and together with the road map, these will be used to make the investment case for prevention, control, and reduction of SBE.

The challenge of building an argument for an NTD that cannot be eliminated, and for which no single universal ‘cure’ is available, is substantial. But the reality is that there is good evidence demonstrating that effective treatment can dramatically reduce mortality by as much as 85% to 88% and also increase positive healthcare-seeking behaviours [47, 48]. In contrast to some other NTD vectors, venomous snakes cannot be eliminated, but SBE can be effectively prevented and controlled so that the burden of injury and the impact on those affected are substantially reduced. Funding is the only barrier to achieving rapid positive and sustainable change. A strong transformational funding investment from both public and private sectors that addresses the short-, medium-, and long-term needs of delivering effective solutions can ensure that SBE becomes a global public health success story. The WHO strategy of improving the production, quality control, and regulation of these life-saving medicines through a comprehensive program to stimulate modernisation, research and development, and to reinvigorate the market, represents a strong advance on the road to achieving a 50% reduction in global mortality and disability and is a compelling case for such investment. Combined with parallel efforts on community engagement and education, health systems strengthening towards universal healthcare and SDG3, effective partnerships at local, national, regional, and global level, and critically-needed funding, the WHO SBE road map can be transformative and enable many of the world’s poorest and most vulnerable communities to have a chance at living healthy and productive lives.
